# Deep learning health space model for ordered responses

**DOI:** 10.1186/s12911-025-03026-3

**Published:** 2025-05-16

**Authors:** Chanhee Lee, Taesung Park

**Affiliations:** 1 Interdisciplinary Program in Bioinformatics, Seoul National University, Seoul, 08826 Korea; 2 Department of Statistics, Seoul National University, Seoul, 08826 Korea

**Keywords:** Biologically interpretable visualization, Health space model, Deep ordinal neural network

## Abstract

**Background:**

As personalized medicine becomes more prevalent, the objective measurement and visualization of an individual’s health status are becoming increasingly crucial. However, as the dimensions of data collected from each individual increase, this task becomes more challenging. The Health Space (HS) model provides a statistical framework for visualizing an individual’s health status on biologically meaningful axes. In our previous study, we developed HS models using statistical models such as logistic regression model (LRM) and the proportional odds model (POM). However, these statistical HS models are limited in their ability to accommodate complex non-linear biological relationships.

**Methods:**

In order to model complex non-linear biological relationship, we developed deep learning HS models. Specifically, we formulated five distinct deep learning HS models: four standard binary deep neural networks (DNNs) for binary outcomes and one deep ordinal neural network (DONN) that accounts for the ordinality of the dependent variable. We trained these models using 32,140 samples from the Korea National Health and Nutrition Examination Survey (KNHANES) and validated them with data from the Ewha-Boramae cohort (862 samples) and the Korea Association Resource (KARE) project (3,199 samples).

**Results:**

The proposed deep learning HS models were compared with the existing statistical HS model based on the POM. Deep learning HS model using DONN demonstrated the best performance in discriminating health status in both the training and external datasets.

**Conclusion:**

We developed deep learning HS models to capture complex non-linear biological relationships in HS and compared their performance with our previously best-performing statistical HS model. The deep learning HS models show promise as effective tools for objectively and meaningfully visualizing an individual’s health status.

**Clinical trial number:**

Not applicable.

## Background

As technology advances, the collection of personal health-related data, such as electronic health records (EHRs), fitness tracker data, and multi-omics datasets, is becoming cheaper and easier [1]. Utilizing this data, comprehensive and continuous monitoring of individual health conditions is expected to be possible, ultimately contributing to the advancement of personalized medical treatments [2]. However, as the dimensions of data that can be collected from an individual increase, visualizing an individual’s health status becomes more challenging [3]. For instance, today, we measure high-dimensional multi-omics datasets, including genomics, transcriptomics, proteomics, and epigenomics, for more precise diagnosis and prognosis [4]. The high dimensionality of these datasets makes it difficult to present multiple types of health-related information in a meaningful and understandable way to both patients and their physicians. Therefore, developing effective data visualization methods that can integrate and display these complex datasets will be essential for clinicians and patients to make clear, evidence-based decisions about personalized treatment options.

For visualization of high-dimensional data, numerous dimensional reduction techniques have been developed in the fields of statistics and machine learning [5]. For linear approaches, principal component analysis is widely used, while non-linear techniques such as autoencoders, t-distributed stochastic neighbor embedding, and uniform manifold approximation and projection are applied across various fields [5–7]. However, the axes generated by these dimensional reduction techniques are often difficult to interpret biologically, as most methods focus on statistical aspects of the data, such as finding optimal linear or non-linear combinations of features that maximize variance or preserve local or global structures. Although these axes may meet optimal statistical criteria, they often lack biological meaning and do not provide useful insights into the underlying biological processes [8].

To address the issue of biological interpretability, a statistical method called ‘Health Space (HS)’ was developed. HS summarizes high-dimensional health data into a compact and visualizable form [8]. HS provides a statistical framework for visualizing an individual’s current health status based on predefined axes that are parameterized in a biologically meaningful way [8]. There are several benefits to using HS for visualizing health status, with the primary advantage being the biological interpretability of the axes. In the original development of HS, three axes were selected to represent overarching processes of human health: oxidation, metabolism, and inflammation [8, 9]. Large values on these three axes indicate that the individual has high levels of oxidative stress, metabolic stress, and inflammation, suggesting a significant deviation from normal health conditions. Conversely, low values suggest that the individual is in a healthy state. These axes are biologically meaningful, representing the relative health status of an individual. Therefore, HS models allow researchers to analyze and interpret the trajectory of an individual’s health status. These models enable continuous monitoring of health conditions and can be used to analyze the effects of treatment in randomized controlled trials. Additionally, the HS models are highly flexible and can be constructed using various statistical models, such as logistic regression model (LRM), proportional odds model (POM), linear mixed-effects model (LMM), and machine learning algorithms [10–12].

An important aspect in building useful HS is to retain as much information as possible when reducing the high-dimensional data into two or three dimensions [13]. The better HS models, the more accurately they differentiate between healthy and diseased cohorts. HS models can be constructed with many different types of statistical models, raising the question of how to choose the optimal HS model. In our previous work, we developed a novel measure called Health Space Index (HSI) to evaluate HS models. The HSI ranges from zero to one, with lower values indicating better model performance [14]. We tested three different HS models based on LRM, LMM, POM and showed POM performed the best [14].

HS models using POM have the advantage of utilizing the ordinal information of the dependent variables, but they lack flexibility in modeling non-linear relationships between the phenotype and independent variables. This can be attributed to the fact that systematic component of the model assumes linear combinations of independent variables, as is a common assumption in statistical modeling. Deep learning, however, has demonstrated strong performance in areas where data is high-dimensional and the relationships between dependent and independent variables are complex and non-linear [15]. While current statistical HS models offer benefits such as the ability to calculate confidence intervals and perform statistical tests on fitted coefficients, they may not be sufficient to model complex non-linear biological processes.

In this study, we propose deep learning HS models that are expected to capture complex non-linear biological processes better than traditional statistical HS models. We initially consider deep learning HS models utilizing standard binary deep neural network (DNN), which collapse ordinal phenotype information into binary phenotype categories. However, these binary DNN models do not effectively account for the ordinal nature of the phenotype. To address this limitation, we propose a deep learning HS model based on a deep ordinal neural network (DONN) that preserves the ordinality of the dependent variable [16]. Through empirical studies using three datasets, we compared the new deep learning HS models—both binary DNNs and DONN—with the statistical HS model based on the POM. The results showed that the deep learning HS model using DONN outperformed the others.

## Methods

### Data descriptions

Three types of data were used in this study. The first dataset was used for training the models, while the other two datasets were used for external validation. For training the HS models, we utilized data from the Korea National Health and Nutrition Examination Survey (KNHANES) 2007 − 2016, which included 32,140 samples [16]. The two external datasets used for model validation were the Ewha–Boramae cohort, a medical examination dataset with 862 samples [17]; and a subset of the Korean Association Resource (KARE) project, a population-based cohort study, with 3,199 samples [18].

To express health status as ordered data set, we split the individuals into four groups in our analysis: First group is healthy group (labeled as 0), second group is a group with one metabolic risk factor (labeled as 1), third group is a group with two metabolic risk factors (labeled as 2), and the fourth group is a group with metabolic syndrome or oxidative stress-related disease group (labeled as 3). The fourth group is associated with oxidative and metabolic stress and is defined based on the presence of any of the following diseases [19–24]: metabolic syndrome, diabetes mellitus, dyslipidemia, severe obesity, intermediate coronary syndrome, stroke, hypertension, and diet-related cancers (liver, colon, stomach, breast, prostate, and lung).

We constructed the HS model using two axes: oxidative stress and metabolic stress. Variables associated with each axis were selected based on their biological relevance. For the oxidative stress axis, we used variables such as age, sex, smoking status, white blood cell count, and alanine aminotransferase levels. For the metabolic stress axis, we included age, sex, body mass index, triglycerides, high-density lipoprotein cholesterol, and fasting glucose levels. Detailed information on the datasets can be found in the methods section of our previous study [14].

### Deep learning health space model using deep ordinal neural network

The HS model outputs a vector in two dimensions where the input is a $\:p$-dimensional vector from a single sample. The first value, corresponding to the oxidation score, ranges from 0 to 1. As the oxidation score increases, the individual’s health status worsens concerning oxidative stress. The second value, corresponding to the metabolism score, also ranges from 0 to 1. As the metabolism score increases, the individual’s health status deteriorates in terms of metabolic stress. Given $\:n$ samples with $\:p$ covariates, the HS model outputs an $\:n\times\:2$ matrix, which can be visualized in a two-dimensional plot. Healthy individuals are typically located in the lower left region of the plot, as they generally exhibit low oxidation and metabolism scores. Conversely, individuals with metabolic syndrome or oxidative stress-related diseases are found in the upper right region, while those with one or two risk factors are positioned between these two groups.

We developed deep learning HS model using DONN which can handle multiple categories with ordinal information. DONN was motivated by the idea from the neural network structure used for ordinal regression [25]. Let $\:\varvec{X}$ be a $\:n\times\:p$ matrix of $\:n$ samples with $\:p$ covariates and $\:\varvec{Y}$ be $\:n\times\:1$ vector where each element can take values from $\:\{\text{0,1},\dots\:,J-1\}$ representing ordinal response with $\:J$ categories. We define a non-linear function $\:f\::\:{\mathbb{R}}^{n\times\:p}\to\:\:{\mathbb{R}}^{n\times\:q}\:$ that maps ***X***(independent variables) with multiple hidden layers and non-linear activation function resulting in $\:\varvec{W}=f\left(\varvec{X}\right).\:$Let $\:{\varvec{X}}_{i}$ and $\:{\varvec{W}}_{i}$ be *i*th row of $\:\varvec{X}$ and $\:\varvec{W}$ respectively and $\:{\varvec{Y}}_{\varvec{i}}$ be *i*th element of $\:\varvec{Y}$. Deep learning HS model using DONN model can be represented as

$$\:\text{log}\frac{\text{Pr}\left({\varvec{Y}}_{\varvec{i}}>j|{\varvec{X}}_{\varvec{i}}\right)}{\text{Pr}\left({\varvec{Y}}_{\varvec{i}}\le\:j|{\varvec{X}}_{\varvec{i}}\right)}={\alpha\:}_{j}+{\varvec{W}}_{\varvec{i}}^{\varvec{\top\:}}\varvec{\beta\:},$$

where $\:\text{Pr}\left({\varvec{Y}}_{i}\le\:j|{\varvec{X}}_{i}\right)\:$is cumulative probability of for $\:i$th sample belonging in categories less or equal to $\:j\in\:\left\{\text{0,1},\dots\:,J-2\right\}$, $\:{\alpha\:}_{j}\in\:\mathbb{R}$ and $\:\varvec{\beta\:}\in\:{\mathbb{R}}^{q}$ are intercepts and coefficients for $\:\varvec{W}$ respectively. We define $\:i$th individual’s health score for DONN to be $\:{\varvec{W}}_{\varvec{i}}^{\varvec{\top\:}}\varvec{\beta\:}$. We fit the model twice, using covariates relevant to oxidative stress, $\:{\varvec{X}}_{oxi}\in\:{\mathbb{R}}^{n\times\:{p}_{1}}\:$, and then using covariates relevant to metabolic stress, $\:{\varvec{X}}_{meta}\in\:{\mathbb{R}}^{n\times\:{p}_{2}}$. Finally, oxidation scores and metabolism scores are calculated for $\:n$ samples as ($\:{\widehat{\varvec{W}}}_{oxi}{\widehat{\varvec{\beta\:}}}_{oxi}$, $\:{\widehat{\varvec{W}}}_{meta}{\widehat{\varvec{\beta\:}}}_{meta})\in\:{\mathbb{R}}^{n\times\:2}$. Note that $\:\widehat{\varvec{W}}=\widehat{f}\left(\varvec{X}\right)$ is also estimated during the fitting process of DONN as parameters in hidden layers are fitted. For both training dataset and validation datasets we set $\:J=4$, $\:{p}_{1}=5$, and $\:{p}_{2}=6.$

**Fig. 1:**
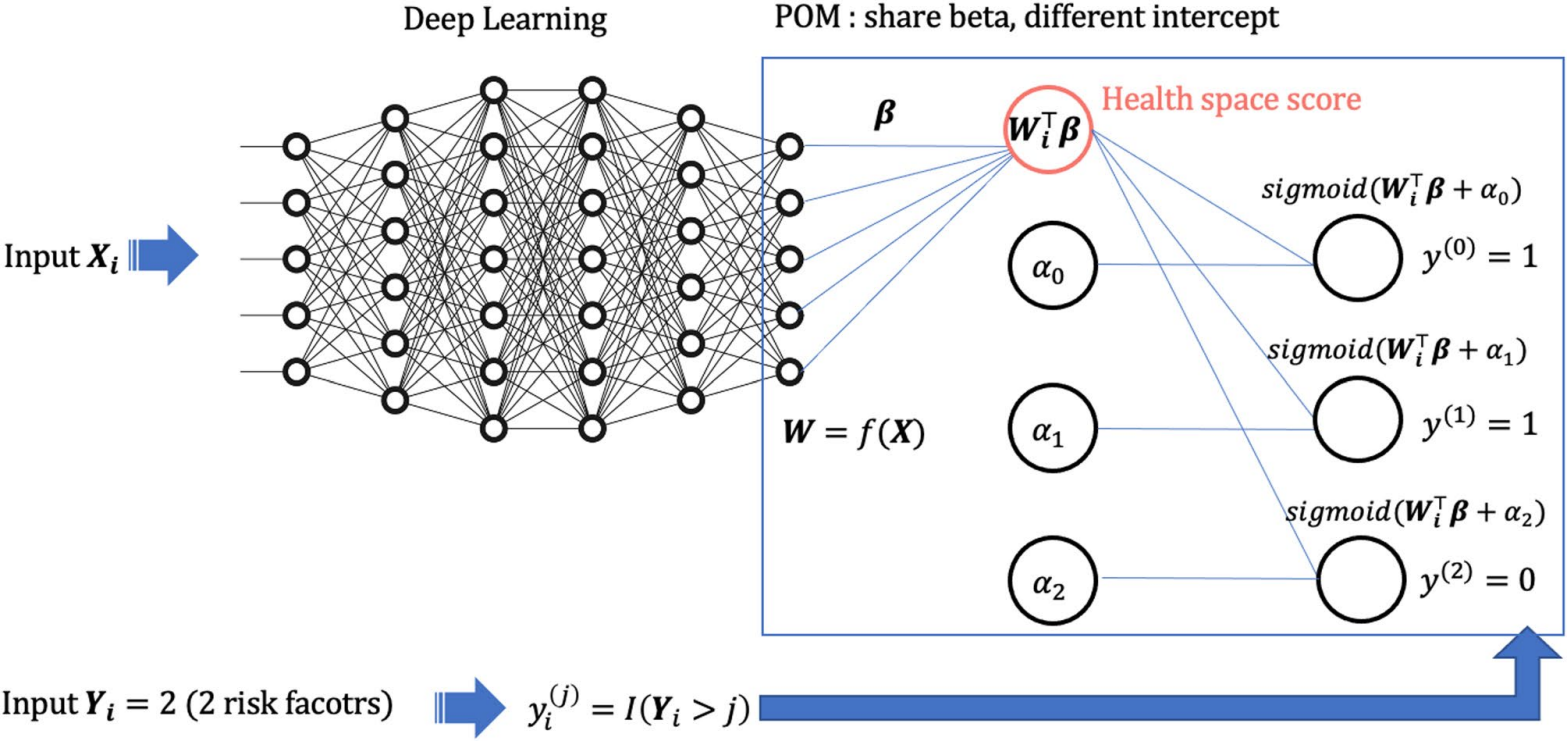
Model structure of deep learning HS model using DONN DONN can handle ordinal data in the response by using idea from POM. It shares beta coefficients but have different intercepts. Also, it utilizes the advantages from deep learning which can model non-linearity between independent variables and the response. This figure illustrates the case when the ordinal response is 2

Loss function of DONN is defined using cross-entropy of $\:J-1$ binary classifiers as

$$\begin{aligned}\:-{{\Sigma\:}}_{i=1}^{n}{{\Sigma\:}}_{j=0}^{J-2}&\{\text{log}\left(\sigma\:(f\left({x}_{i}\right)+{\alpha\:}_{j}\right){y}_{i}^{\left(j\right)}\\&+\text{log}\left(1-\sigma\:\left(f\left({x}_{i}\right)+{\alpha\:}_{j}\right)\right)(1-{y}_{i}^{\left(j\right)})\},\end{aligned}$$

where $\:\sigma\:(\cdot\:)\:$is sigmoid function and $\:{y}_{i}^{\left(j\right)}=I\left({Y}_{i}>j\right)$ with indicator function $\:I(\cdot\:)$. For optimization, Adam optimizer was used and learning rate was set to 0.001 [26]. For hyperparameters settings used in DONN, ReLU function was used for non-linear activation function, and number of hidden layers were set to two [27]. Batch size was set to 100, and training epochs were set to 150 with early stopping. Implementation of the code was done using PyTorch (version 1.21.1) package [28] in Python (version 3.8.16). Figure 1 summarizes the model structure of deep learning HS model using DONN.

### Deep learning health space models using binary deep neural network

We also considered four different deep learning HS models using binary DNN. First, we considered simple binary DNN model (0 vs. 3) for HS focusing only in 0 and 3 categories. In this model, we only included data with outcome response of 0 and 3, excluding those with responses of 1 and 2. Using the same notation from the previous section of DONN, the model can be written as

$$\:\text{log}\frac{\text{Pr}\left({\varvec{Y}}_{\varvec{i}}=3|{\varvec{X}}_{\varvec{i}}\right)}{\text{Pr}\left({\varvec{Y}}_{\varvec{i}}=0|{\varvec{X}}_{\varvec{i}}\right)}=\alpha\:+{\varvec{W}}_{\varvec{i}}^{\varvec{\top\:}}\varvec{\beta\:}.$$

HS scores for this binary DNN (0 vs. 3) can be calculated as ($\:{\widehat{\varvec{W}}}_{oxi}{\widehat{\varvec{\beta\:}}}_{oxi}$, $\:{\widehat{\varvec{W}}}_{meta}{\widehat{\varvec{\beta\:}}}_{meta})\in\:{\mathbb{R}}^{n\times\:2}$. Next, we considered three binary DNN models for HS that can utilize all the data by collapsing the ordinal response into binary response, i.e., (0 vs. 1 + 2 + 3), (0 + 1 vs. 2 + 3), and (0 + 1 + 2 vs. 3). These models can be summarized as

$$\:\text{log}\frac{\text{Pr}\left({\varvec{Y}}_{\varvec{i}}>j|{\varvec{X}}_{\varvec{i}}\right)}{\text{Pr}\left({\varvec{Y}}_{\varvec{i}}\le\:j|{\varvec{X}}_{\varvec{i}}\right)}={\alpha\:}_{j}+{\varvec{W}}_{j,i}^{\varvec{\top\:}}{\varvec{\beta\:}}_{j},$$

where $\:j\in\:\left\{\text{0,1},2\right\}$. Note that we have three separate models where $\:{\varvec{W}}_{j,i}^{\varvec{\top\:}}$ is the *i*th row vector of *j*th $\:\varvec{W}$. HS scores for collapsed binary DNN with combined categories can be calculated as $\:{\widehat{(\varvec{W}}}_{j,oxi}{\widehat{\varvec{\beta\:}}}_{j,oxi}$, $\:{\widehat{\varvec{W}}}_{j,meta}{\widehat{\varvec{\beta\:}}}_{j,meta})\in\:{\mathbb{R}}^{n\times\:2}$ for $\:j\in\:\{\text{0,1},2\}$. Optimization algorithm and hyper parameters were set to be same with DONN to remove bias on the performance HS models with regard to differences in hyper-parameters.

### Evaluation measures of Heath space models

Since the primary objective of the HS model is to provide a biologically meaningful visualization rather than classification, standard classification metrics such as AUC, accuracy, recall, and precision are not suitable for comparing HS models. To evaluate and compare HS models, we used the Health Space Index (HSI), which is specifically designed for this purpose [14]. HSI assesses the separation between different health status groups in $\:n$-dimensional space using confidence ellipses and the Jaccard index, making it highly flexible and generalizable. HSI can be applied to any number of groups and axes, providing a general approach for evaluating HS model. We slightly adjusted the original definition of HSI to 1 - HSI, so that higher HSI values indicate better-performing HS models.

In addition to HSI, we considered three additional measures to enhance the objectivity and comprehensiveness of the evaluation process. First, the Silhouette Score was used, which measures how similar an object is to its own cluster compared to other clusters [29]. It ranges from − 1 to 1, with higher values indicating better-clustered objects and better clustering quality. In the context of HS models, a high Silhouette Score suggests that the health groups are better-separated, indicating a superior HS model. Second, the Davies-Bouldin Index was used, which assesses the average similarity ratio of each cluster to its most similar cluster [30]. It ranges from 0 to infinity, where a lower score indicates better-defined clusters with greater separation between them. For HS models, a lower Davies-Bouldin Index indicates more distinct and better-separated health groups, reflecting a better HS model. Finally, the Calinski-Harabasz Index was used, which evaluates the ratio of the sum of between-cluster dispersion to within-cluster dispersion [29]. This index ranges from 0 to infinity, with higher values indicating better-defined clusters. In evaluating HS models, a higher Calinski-Harabasz Index suggests that the health groups are more compact and better-separated, indicating a better HS model.

## Results

### Comparing HSI between six different HS models in KNHANES data

**Fig. 2:**
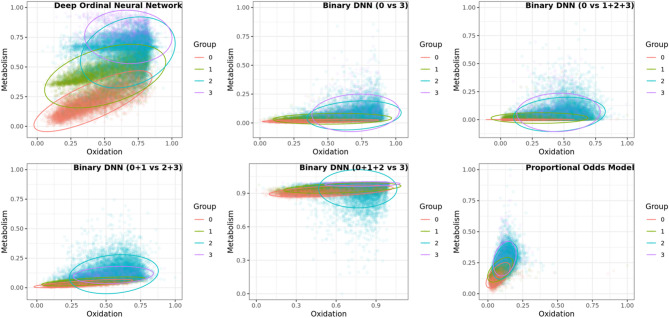
Comparison of HS created by six different HS models using KNHANES dataset

For comparison with our previous statistical HS models, we included our previous best statistical HS model using POM [14]. In summary, six HS models were considered: five deep learning HS models and one statistical HS model using POM. Among the five deep learning HS models, one was DONN and three were binary DNNs created by collapsing the ordinal information into the following categories: (0 vs. 1 + 2 + 3), (0 + 1 vs. 2 + 3), and (0 + 1 + 2 vs. 3). The fifth DNN was developed using partial data, specifically comparing (0 vs. 3). HS plots were created from the different HS models. Traditional machine learning models such as random forests, support vector machines, and XGBoost primarily focus on classification and do not naturally provide the necessary $\:x\beta\:$ (linear predictor) form or continuous scores required for HS visualization. Consequently, these models were not suitable for our framework and were excluded from comparison. Figure 2 summarizes HS models created from six different models using KNHANES data (*n* = 32,140). Good HS models are the ones that discriminate the health status groups better.

**Fig. 3:**
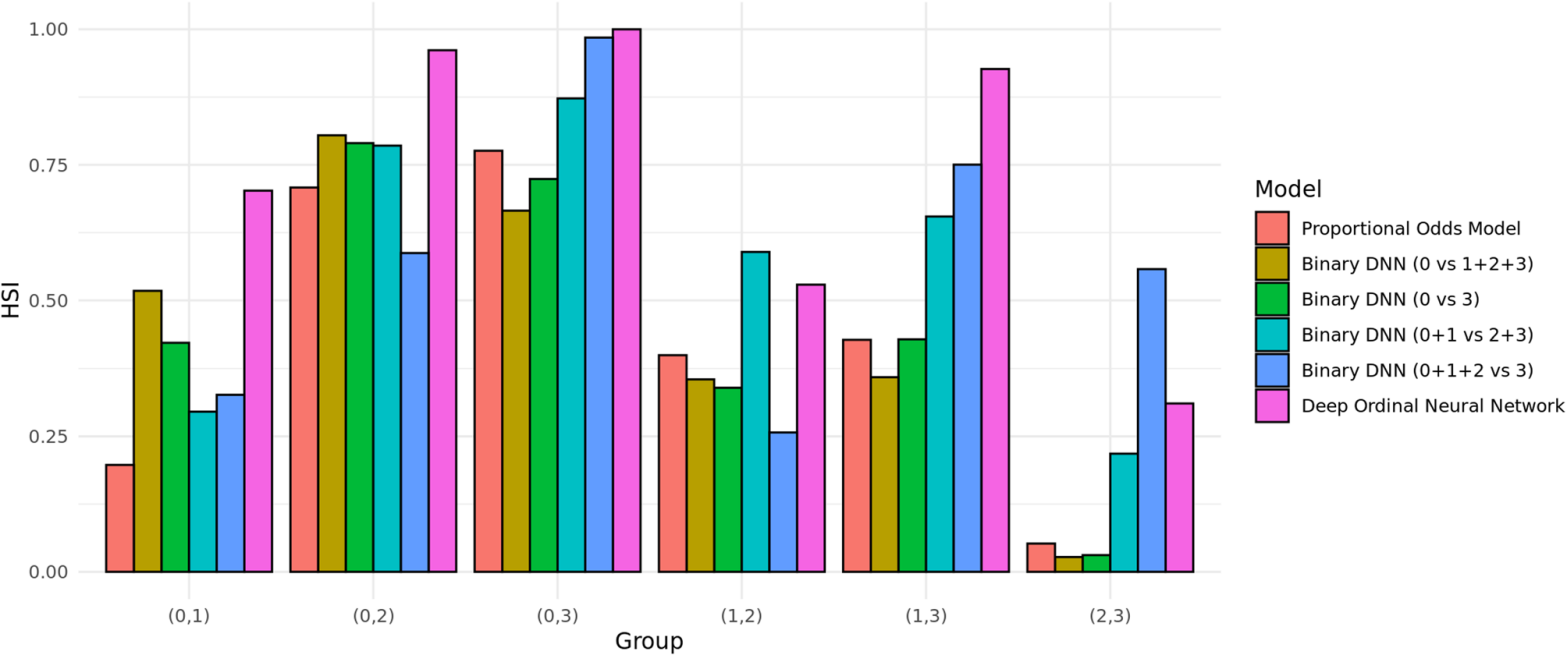
Comparison of pairwise HSI calculated by six different HS models using KNHANES dataset

Even though, HS plot can aid in choosing which HS model is the best, we resort to more objective quantitative measure HSI in evaluating appropriateness of HS models. HSI was originally defined as a measure between two groups. Since we had four groups in total, six pairwise HSIs were calculated for each HS model as shown in Fig. 3. For most of the pairwise HSI, except for (0 vs. 1) and (1 vs. 2), DONN showed highest HSI meaning that it had best separation between the two groups.

**Fig. 4:**
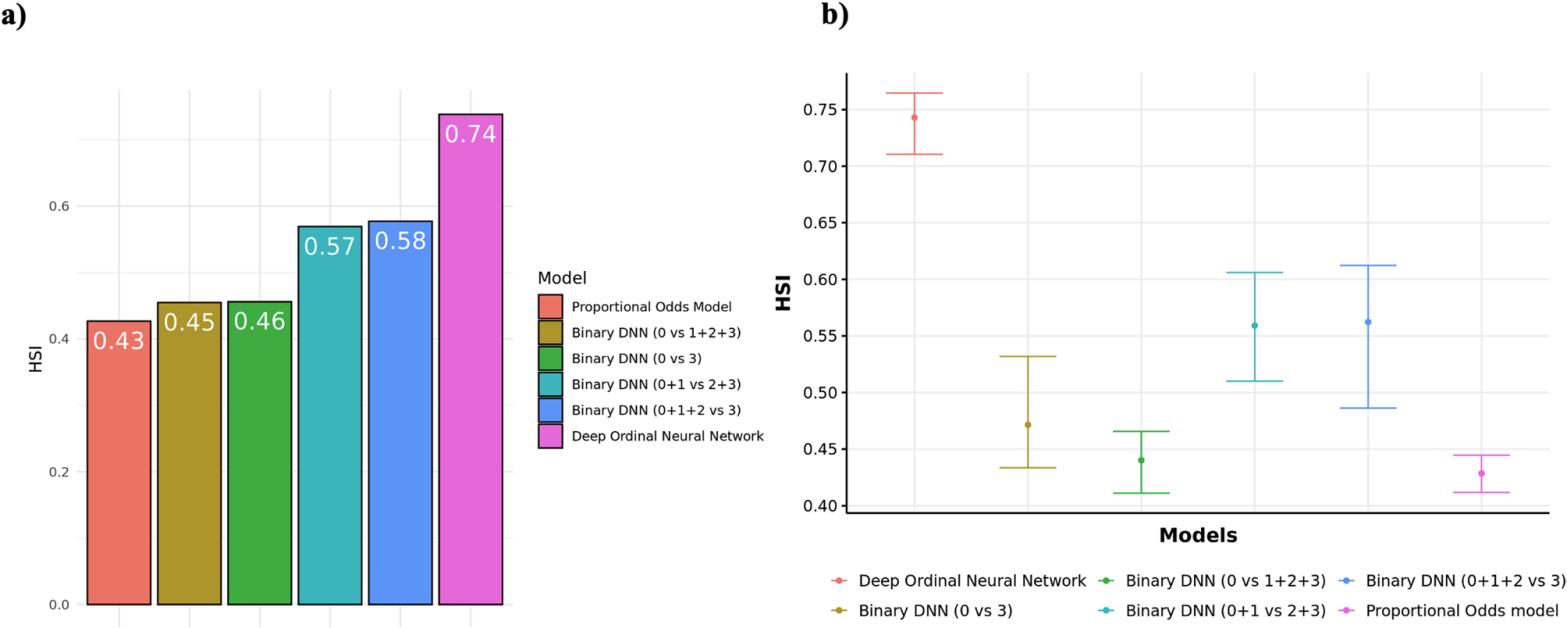
HS models performance comparison using average of HSI using KNHANES dataset. **(a)** Comparing the average HSI across different HS models, showcasing the direct comparison of model performance using KNHANES dataset **(b)** Bootstrapped mean and 95% confidence interval (CI) of the average HSI are compared between HS models. The analysis incorporates 200 bootstrapped samples to ensure a robust estimation of the distribution and to enhance the reliability of the comparison

In order to choose best HS model, we used average of the pairwise HSIs as the measure for comparing HS models, and model with highest HSI was chosen as the best HS model. We calculated average HSI for all six models using KNHANES data. POM showed HSI of 0.43, showing lowest performance compared to other five HS models. Binary DNN models trained with all data by collapsing the label into binary class showed HSI of 0.45 (0 vs. 1 + 2 + 3), 0.57 (0 + 1 vs. 2 + 3) and 0.58 (0 + 1 + 2 vs. 3) respectively. Binary DNN model trained with partial data using healthy group (0) and disease group [3] showed HSI of 0.46. Finally, average HSI of DONN was calculated showing best performance of 0.74. Figure 4a summarizes average HSI of HS models created from six different models. To ensure robustness in our evaluation, we incorporated bootstrapping, which is crucial for accounting for the variability in the training data [31]. Bootstrapping allows us to generate multiple resampled datasets, providing confidence intervals (CI) and assessing the stability and reliability of the model’s performance across different data subsets. The bootstrapped mean of HSI and 95% CI were calculated for all models. The mean HSI for DONN was 0.743, with a 95% CI of (0.710, 0.765), which does not overlap with the CIs of other models. In contrast, the POM had the lowest mean HSI of 0.429, with a CI of (0.412, 0.445). The binary DNN models showed intermediate performance with mean HSIs ranging from 0.440 to 0.562, but none overlapped with the DONN’s CI, underscoring robustness and effectiveness of DONN. Figure 4b illustrates the bootstrapped results, including both the bootstrapped mean and the bootstrapped 95% confidence interval for 200 bootstrapped samples.

### Comparing HS models in external validation data

**Fig. 5:**
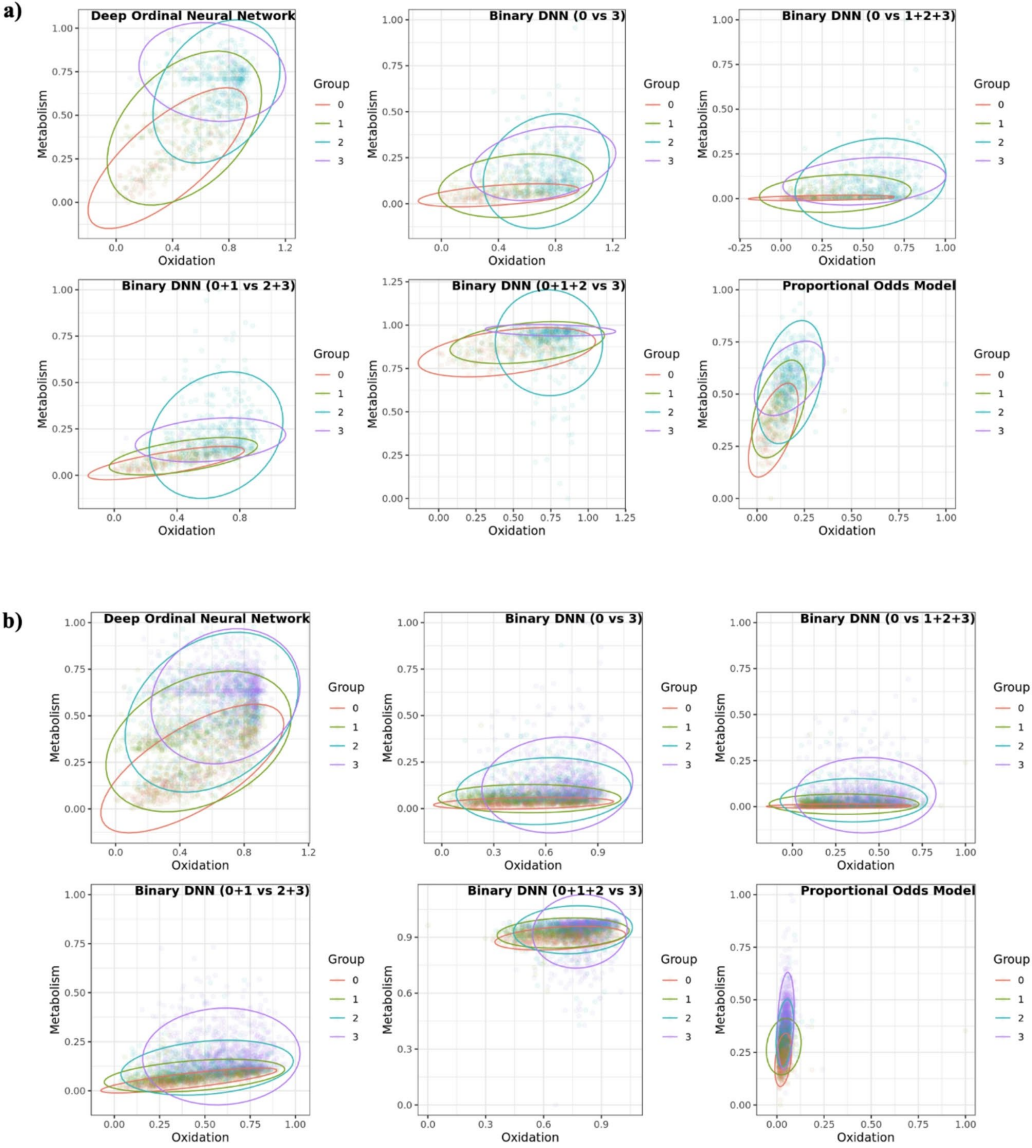
Comparison of HS created by six different HS models in external dataset. **(a)** HS plot of Ewha-Boramae datasets ($\:n=862$) **(b)** HS plot of KARE datasets ($\:n=\text{3,199}$)

To test the general performance of HS models, we used two external validation datasets, Ewha-Boramae cohort data (*n* = 862) and KARE cohort data (*n* = 3,199). These two external datasets were not included in the training process, as only KNHANES (*n* = 32,140) was used for that purpose. Figure 5 summarizes HS models created from six different models using Ewha-Boramae cohort data (Fig. 5a) and KARE cohort data (Fig. 5b). The deep learning HS model using DONN demonstrated superior performance across both external datasets, achieving the highest HSI, with scores of 0.53 in Ewha-Boramae and 0.41 in KARE, ranking first in both datasets. DONN also excelled in clustering measures, recording the highest average Silhouette Score (-0.08 in Ewha-Boramae, and 0.00 in KARE) and the highest Calinski-Harabasz Index (487.60 in Ewha-Boramae, 1475.77 in KARE), again ranking first. For the Davies-Bouldin Index, DONN showed the lowest value of 2.41 for Ewha-Boramae and the third-lowest value of 3.34 for KARE, ranking first and third respectively.

In contrast, the deep learning HS models using binary DNN demonstrated varied performance across the datasets. The binary DNN (0 vs. 3) model ranked fifth in HSI in Ewha-Boramae (0.43) and second in KARE (0.40). In terms of Silhouette Score, it ranked fourth in Ewha-Boramae (-0.21) and fifth in KARE (-0.15). For the Calinski-Harabasz Index, it ranked fourth in Ewha-Boramae (121.16) and fourth in KARE (399.87). Lastly, for the Davies-Bouldin Index, it ranked second in Ewha-Boramae (4.37) and fifth in KARE (4.42). The statistical HS model using POM performed consistently across most measures, ranking second overall, except in HSI for Ewha-Boramae (0.47, 4th) and the Davies-Bouldin Index (9.67, 5th). Overall, the DONN model consistently outperformed the other models across almost all measures and datasets, demonstrating its superiority in clustering quality and overall model effectiveness. The results are summarized in Table 1.

**Table 1:** HS models performance evaluation in external validation datasets

	KNHANES(*n* = 32,140)				Ewha-Boramae (*n* = 862)				KARE (*n* = 3,199)			
	Average Health space index	Average Silhouette score	Calinski-Harabasz index	Davies Bouldin index	Average Health space index	Average Silhouette score	Calinski-Harabasz index	Davies Bouldin index	Average Health space index	Average Silhouette score	Calinski-Harabasz index	Davies Bouldin index
Deep Ordinal Neural Network	**0.74**	**0.09**	**49722.82**	**1.66**	**0.53**	**-0.08**	**487.60**	**2.41**	**0.41**	**0.00**	**1475.77**	3.34
Binary DNN (0 vs. 3)	0.46	-0.04	4988.67	4.37	0.43	-0.21	121.16	4.16	0.40	-0.15	399.87	4.42
Binary DNN (0 vs. 1 + 2 + 3)	0.45	-0.08	3386.12	5.83	0.42	-0.28	74.70	9.57	0.36	-0.26	267.53	4.3
Binary DNN (0 + 1 vs. 2 + 3)	0.57	-0.10	9693.17	10.59	0.51	-0.20	157.85	5.45	0.38	-0.10	759.1	**2.68**
Binary DNN (0 + 1 + 2 vs. 3)	0.58	-0.18	2681.57	8.17	0.49	-0.29	62.25	13.56	0.29	-0.11	141.49	13.59
Proportional Odds Model	0.43	-0.04	19134.58	5.35	0.47	-0.10	319.78	9.67	0.40	-0.02	882.99	3.19

The performance of HS models is compared using the average HSI, average Silhouette Score, Davies-Bouldin Index, and Calinski-Harabasz Index. All the models were trained using the KNHANES dataset and validated on two external datasets, the Ewha-Boramae and KARE datasets. The best-performing HS model for each measure is highlighted in bold

## Discussion

As the dimensions of health-related data for individuals continue to increase, it is becoming increasingly important to measure and visualize an individual’s health status in an objective and biologically interpretable way. One effective statistical method for visualizing an individual’s health status is the HS model. The HS model maps an individual’s health status into two dimensions using axes that represent overarching processes of human health, enabling clear visualization and interpretation. In this study, we developed deep learning HS models capable of flexibly modeling non-linearity. Among these, the DONN, which utilizes ordinal information, achieved the highest HSI compared to other binary DNN HS models. It also outperformed our previous statistical HS model based on the POM. Bootstrapped results showed that the 95% confidence interval for DONN did not overlap with any other HS models. Through empirical studies using external validation datasets, we demonstrated that deep learning HS model utilizing DONN outperforms other HS models across various measures.

We found that when applying deep learning to HS models, it’s essential to consider the data’s underlying structure, such as ordinality. Simply applying conventional neural networks without this consideration can lead to suboptimal results. Improved deep learning HS models allow for more accurate separation of individuals by health status. This enhances the power of downstream analyses, including the precision of health intervention evaluations. In well-crafted HS models capable of detecting subtle distinctions in health status, the effects of dietary adjustments and other interventions can be identified with greater precision. Moreover, these models can also help reveal individual patterns in health trajectories, allowing for the identification of subgroups with distinct responses to interventions. To formally identify these subgroups, clustering analysis methods can be applied to trajectory data, grouping individuals based on the similarity of their health trajectories within the HS space. By tracking changes in an individual’s position within the HS space over time, HS models can provide insights into variations in response patterns, facilitating more personalized and targeted healthcare strategies. Furthermore, if sufficient trajectory data is available and the progression patterns of specific diseases are well-characterized, HS models could be utilized to predict future health outcomes. To achieve this, a prediction model can be constructed by incorporating trajectory data within the HS space. First, by tracking an individual’s movement within the HS space over time, patterns of health progression can be quantified. Using these trajectories, time-series modeling techniques can be applied to forecast future positions in the HS space. By analyzing an individual’s movement within the HS space, HS models may help identify those at high risk of developing certain diseases, enabling early interventions and more effective risk stratification.

The deep learning HS models presented in this study offer significant advancements in health status visualization, but some limitations should be considered. First, interpretability remains a challenge, as deep learning models, including DONN, do not inherently provide feature-level explanations. Unlike statistical models such as POM, where *β* coefficients directly indicate feature importance, deep learning models lack transparency, making it difficult to pinpoint the biological factors driving individual placements within the HS space. Second, while our model is currently designed around oxidative and metabolic stress axes, these two dimensions do not fully capture the complexity of human health. Third, generalizability and potential overfitting remain concerns, as the model was trained on large but Korean-specific datasets, which may limit its applicability to populations with different genetic, environmental, and lifestyle factors. Finally, the computational complexity of deep learning models may pose challenges for widespread adoption in clinical settings.

For future research, several promising directions could further enhance the impact and applicability of HS models. First, improving model interpretability is crucial. Applying explainable AI (XAI) techniques such as Shapley additive explanations (SHAP), attention mechanisms, or feature attribution methods could help elucidate the biological factors influencing health status visualization [32, 33]. Second, expanding the HS framework beyond oxidative and metabolic stress by incorporating additional axes, such as inflammation or cardiovascular health, could improve its biological relevance and provide a more holistic representation of health status [34, 35].This can be achieved by selecting biologically meaningful markers from multi-omics data with corresponding phenotype collection, fitting the DONN model to capture non-linear relationships, and integrating the new axes with the existing ones. Third, to improve generalizability, future studies should focus on validating HS models in multi-ethnic cohorts and assessing whether overfitting to the Korean cohort has occurred. Finally, translating HS models into clinical practice remains an open challenge. Evaluating their usability through prospective studies or pilot clinical implementations will be critical for determining their impact on clinical decision-making and patient outcomes [36]. Addressing these areas will further strengthen the interpretability, generalizability, and clinical utility of HS models, advancing their role in personalized medicine and health visualization strategies.

## Conclusions

The objective of this study is to build a simple and effective visualization methodology that can help easily recognize the health status of individuals. Our newly developed deep learning HS models can handle non-linearity in the data and are based on oxidative and metabolic stresses, making them more biologically interpretable. Among the developed deep learning HS models, the DONN, which handles ordinal data, performed the best compared to other HS models. Our improved HS models facilitate the visualization of complex biological data in a two-dimensional plot and demonstrate the usefulness of incorporating deep learning in HS modeling.

## Data Availability

The KNHANES and KARE datasets can be provided after review and evaluation of research plan by the Korea Centers for Disease Control and Prevention (http://www.cdc.go.kr/CDC/eng/main.jsp). The Ewha-Boramae dataset is available from the corresponding author on reasonable request.

## Abbreviations

HS: Health Space
HSI: Health Space Index
DNN: Deep neural network
LRM: Logistic regression model
LMM: Logistic mixed effects model
POM: Proportional odds model
DONN: Deep ordinal neural network
KARE: Korea Association Resource
KNHANES: Korea National Health And Nutrition Examination Survey
